# Genetic Polymorphisms of Prokineticins and Prokineticin Receptors Associated with Human Disease

**DOI:** 10.3390/life14101254

**Published:** 2024-10-01

**Authors:** Roberta Lattanzi, Rossella Miele

**Affiliations:** 1 Department of Physiology and Pharmacology “Vittorio Erspamer”, Sapienza University of Rome, Piazzale Aldo Moro 5, I-00185 Rome, Italy; 2 Department of Biochemical Sciences “A. Rossi Fanelli”, CNR-Institute of Molecular Biology and Pathology, Sapienza University of Rome, Piazzale Aldo Moro 5, I-00185 Rome, Italy

**Keywords:** prokineticins, prokineticin receptors, G-protein-coupled receptors, polymorphisms

## Abstract

Prokineticins (PKs) are low molecular weight proteins that exert their effects by binding to two seven-transmembrane G-protein-coupled receptors (prokineticin receptors, PKRs). The prokineticin system is an important player in the development of various diseases. Several polymorphisms that are associated with infertility, neuroendocrine disorders, Hirschsprung’s syndrome (HSCR), idiopathic central precocious puberty (CPP) and congenital disorders such as Kallmann syndrome (KS) have been described for both the PKs and PKR genes. The aim of this study is to summarize and describe the impact of PK/PKR polymorphisms on the pathogenesis and outcome of the above diseases, highlighting the PK system as a therapeutic target and diagnostic biomarker in pathological conditions.

## 1. Introduction

The prokineticin family comprises two ligands, prokineticin 1 (PK1) and prokineticin 2 (PK2), and two G-protein-coupled receptors (GPCRs) called prokineticin receptor 1 (PKR1) and prokineticin receptor 2 (PKR2) [1,2,3,4,5]. The prokineticin system is modulated by the binding of accessory proteins such as b-arrestin, Melanocortin 2 Receptor Accessory Protein 2 (MRAP2) and Snapin [5,6,7,8,9]. Prokineticin signalling is involved in several important physiological functions, including contraction of gastrointestinal smooth muscle, regulation of circadian rhythm, neurogenesis and regulation of mood and reproduction [4,5]. Dysregulation of prokineticin signalling has been observed in a number of diseases, such as inflammation, neurodegeneration, cancer and obesity, where PKs/PKRs appear to be promising therapeutic targets [4,10,11,12,13].

In humans, the genes encoding PK1 and PK2 are located on two different chromosomes: the PK1 gene [Online Mendelian Inheritance in Man (OMIM) * 606233] is located on chromosome 1 and the PK2 gene (OMIM * 607002) has been identified in an unstable chromosomal region near a synthetic breakpoint on chromosome 3 [14]. The structure of the two genes is different, in particular, the PK2 gene has four different exons, whereas the PK1 gene has only three exons. The greater complexity of the PK2 gene allows the existence of alternative splicing mechanisms that lead to the production of three different isoforms, PK2, PK2b and PK2c [5,15,16,17]. In humans, the genes coding for prokineticin receptors are located on two different chromosomes: the PKR1 gene (OMIM * 607122) is located on chromosome 2 (2p13.3) and the PKR2 gene (OMIM * 607123) is on chromosome 20 (20p13) and consists of three exons and two introns [18]. The first exon contains a 5′ untranslated region (UTR); the second exon contains part of the 5′ UTR sequence and a region encoding the first three transmembrane domains TM1, TM2 and TM3; and the third exon encodes the last transmembrane domains (TM4, TM5, TM6 and TM7) and the 3′ UTR sequence. The second intron is located at the TM3 boundary within the common DRY (Asp-Arg-Tyr) sequence. Two PKR2 isoforms are generated from the PKR2 gene by alternative splicing: PKR2 and TM4-7 [19].

**Figure 1:**
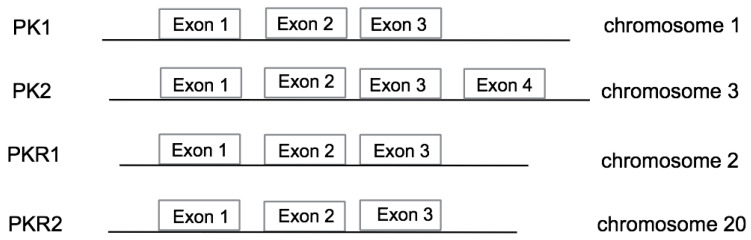
Schematic representations of the human PK1 (gene ID: 84432), PK2 (gene ID: 60675), PKR1 (gene ID: 10887) and PKR2 (gene ID: 128674) gene structures.

Several polymorphisms have been described for both the PK and PKR genes (Figure 1). The polymorphism in the PK1 gene is associated with pathologies related to fertility and reproduction, while the polymorphism in the PK2 gene is mainly associated with neuroendocrine disorders. The polymorphisms in the PKR1 gene are associated with recurrent pregnancy loss and Hirschsprung’s syndrome (HSCR), a syndrome in which the organisation of the ganglia of the nervous system is altered during embryogenesis. The localization of the PKR2 gene in a region that is highly susceptible to mutations justifies the existence of several polymorphisms of the PKR2 gene that are associated with some diseases. The PKR2 polymorphisms have been detected in idiopathic and recurrent pregnancy loss, in depressive and bipolar disorders and in methamphetamine addiction. More recently, additional polymorphisms have been found in idiopathic central precocious puberty (CPP) and in congenital disorders such as Kallmann syndrome.

This review focuses on some of the latest findings related to prokineticin polymorphisms with the aim of investigating the genetic polymorphisms of prokineticins in relation to predisposition, pathogenesis and outcome of human diseases [4,5,20].

## 2. Disease Associated with Circadian Rhythms

**Figure 2:**
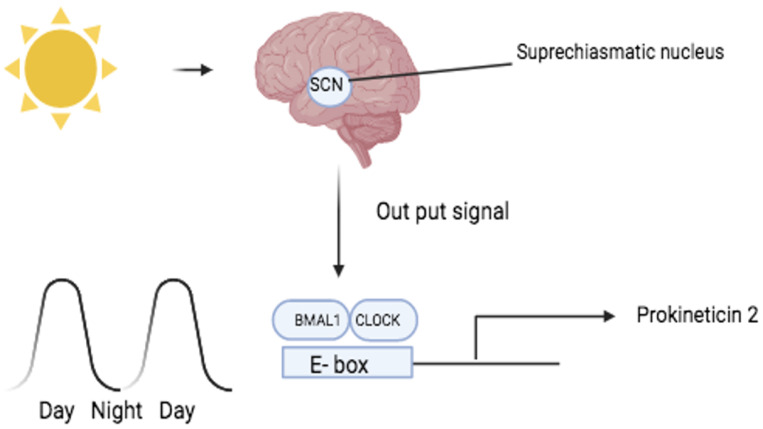
PK2 has been shown to be a candidate output molecule of the suprachiasmatic nucleus (SCN). The molecular PK2 rhythm in the SCN, which consists of strong PK2 expression during the 12 h light phase, is mainly controlled by the transcription factors CLOCK and BMAL, which recognise specific sequences (E-boxes) present in both the human and mouse PK2 promoter [32,33,34].

Circadian rhythm disruption and the resulting sleep–wake alterations are associated with mood disorders such as bipolar (BP) and major depressive disorder (MDD), which have a negative impact on patients’ lives [21,22]. The PK2 gene and the PKR2 gene are involved in the circadian regulation of sleep (Figure 2) and are good candidates for the pathogenesis of mood disorders. Indeed, animal studies have reported that intracerebroventricular injection of PK2 leads to increased anxiety-like behaviour. Conversely, PK2 knockout mice showed significantly reduced anxiety- and depression-like behaviours in the forced swim test and reduced responses to novel environments in terms of locomotor activity, arousal, body temperature and food intake [23]. Both PK2 and PKR2 gene-deficient mice show disturbances in circadian and homeostatic regulation of sleep, such as decreased sleep time with non-rapid eye movement (NREM) and increased sleep time with rapid eye movement (REM) [24]. In addition, PKR2 knockout mice showed impaired circadian coordination of the activity cycle and reduced precision in the timing of the onset of nocturnal locomotor activity [25]. Based on the above considerations, the PK2 and PKR2 genes appear to be good candidates for the pathogenesis of mood disorders. The PK2 and PKR2 genes were located in a susceptibility region for BP in three linkage analysis studies [26,27,28].

To investigate the association between PK2 and PKR2 and mood disorders, Kishi et al. [29] conducted a case–control study in Japanese patients with mood disorders (151 BP patients, 319 patients with severe MDD and 340 control subjects) and found a single nucleotide polymorphism (SNP) in PKR2 that was significantly associated with female BP and MDD [29]. This is because it is known that there are sex differences not only in the pathophysiology of mood disorders [30], but also in circadian rhythms [31]. By demonstrating an association between PKR2 and a female diagnosis of BP or MDD, the authors supported the assumption that the aetiology of mood disorders is different in women and men (Table 1).

## 3. Disease Associated with Psychiatric Disorders

Methamphetamine (METH), a drug in the amphetamine family, is a powerful central stimulant that has become a significant global problem due to its high addictive potential and high mortality [35]. METH is a commonly abused drug in the general population and is often associated with anxiety, depression and even psychosis [36]. Although neurotransmitters such as dopamine [37], glutamate [38] and serotonin [39] have been shown to play a crucial role in the synaptic plasticity of certain brain circuits, the mechanisms of action underlying methamphetamine use disorder (MUD) are not clear and there are no effective drugs for its treatment [40,41]. Therefore, thanks to continuous research in the field with the aim of finding potential new therapeutic targets, Nuclear Factor Erythroid 2-Related Factor 2 (Nrf2), an endogenous protective factor against oxidative stress [42], has been identified as an important player in METH-induced toxicity.

Genetic susceptibility to MUD is poorly understood. Indeed, no twin studies or adequately powered genome-wide association studies (GWAS) have been conducted, although a large number of hypothesis-driven candidate gene association studies have been systematically reviewed in people with MUD [43]. In this study, the authors analysed 75 different genes and calculated allele frequencies. Of the 75 genes analysed in the literature, 29 had at least one marker that showed a significant genotypic or allelic association with MUD, and 14 genes had minor alleles for markers. PKR2 showed the allelic association marker rs6085086, located within an intron, and two minor alleles, rs3746682 and rs4815787, which are located within the 3′ UTR of PKR2 and can modulate gene expression. The three alleles play different roles, with rs3746682 being a protective marker, and rs6085086 and rs4815787 being risk markers.

In a recent paper, Jiang and colleagues [44] investigated the correlation between the PK2 gene and MUD risk and drug craving in 5282 Chinese Han participants (1796 MUD patients and 3486 controls). They selected seven tag SNPs for the PK2 gene, which were genotyped in serum samples. The results showed that there is a genetic variation within the PK2 gene (SNP rs75433452) that is associated not only with susceptibility to MUD risk, but also with craving scores. The SNP rs75433452 is an intronic DNA variant of PK2 whose functional significance is not yet clear. Although the authors were able to demonstrate a significant correlation between this SNP and PK2 serum levels, the data from their Genotype Tissue Expression (GTEx) database indicate that rs75433452 does not show a significant correlation with PK2 expression in any of the 48 tissues analysed.

In summary, this study, which found a significant genetic association between PK2 and MUD risk susceptibility and MUD, is very important because PK2 has the ability to increase the levels of protective factors such as Nrf2 (Table 1).

## 4. Diseases Associated with the Neuroendocrine System

### 4.1. Hypogonadotropic Hypogonadism and Kallmann Syndrome

Hypogonadotropic hypogonadism (HH) is a rare endocrine reproductive disorder that generally leads to an absence of puberty, hypogonadism and infertility. During early embryogenesis, gonadotropin-releasing neurons (GnRHs) originate from the olfactory epithelium, migrate along the axons of the developing olfactory neurons, enter the forebrain, traverse the developing olfactory bulb (OB) and finally migrate to the medial preoptic area of the olfactory epithelium. They position themselves between the OB and the mediobasal hypothalamus. Here, by extending their axons into the median eminence, they release the decapeptide GnRH into the pituitary gland, which influences the synthesis and secretion of gonadotropins from the cells of the anterior pituitary and thus regulates the functions of the gonads [45,46]. Insufficient migration of these neurons leads to a decreased release of pituitary gonadotropins, which is one of the clinical causes of hypogonadotropic hypogonadism (HH). This is characterised by an isolated deficiency of GnRH and delayed or absent puberty. When HH is associated with a reduction or loss of olfactory sensitivity (hyposmia or anosmia), it is referred to as Kallmann syndrome (KS).

**Figure 3:**
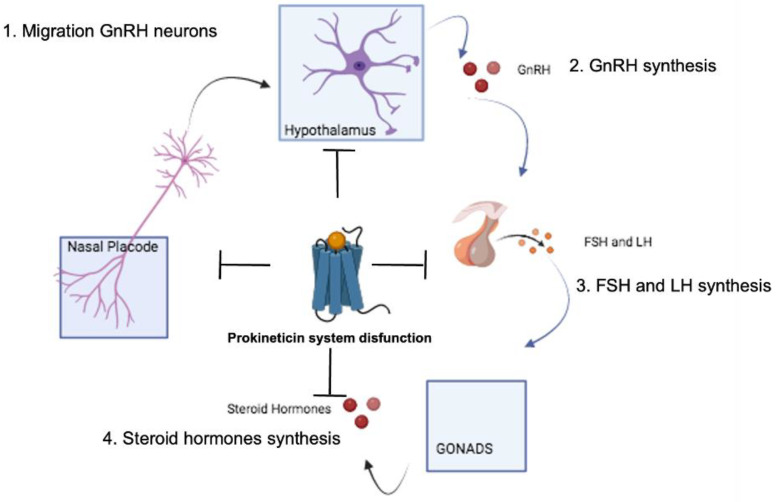
Hypothalamic–pituitary–gonadal axis. The presence of polymorphisms in the PK2 or PKR2 genes determines (1) the absence of neuronal GnRH migration from the nasal placode to the hypothalamus, (2) the absence of GnRH synthesis in the hypothalamus, (3) pituitary dysplasia and reduction in the synthesis of FSH and LH, and (4) hypogonadism and reduction in the synthesis of sex hormones.

Kallmann syndrome is a hereditary disease with complex genetic transmission and hormone replacement therapy is the only treatment that can achieve the development of male and female sexual characteristics and thus fertility of both sexes. Mutations in one of two different genes, KAL1 and FGFR1, have been found in about 20% of affected individuals, and a further 10% of patients have both homozygous and heterozygous mutations in the genes encoding PKR2 or its ligand PK2 [47,48] (Figure 3). Remarkably, some of the PK2 and PKR2 mutations have also been detected in individuals who are not clinically affected by the disease, clearly suggesting that additional genetic or non-genetic factors are involved in the development of KS. In 2010, Sykiotis et al. analysed eight HH-associated genes, including ANOS1, GNRHR, KISS1R, FGFR1 and WDR11, and showed that 11.3% of HH patients had more than one genetic mutation. Nine of 18 patients with PKR2 mutations carried 1–2 rare mutations in other HH-associated genes, and the HH phenotype may be an additive effect of multiple mutations [49,50,51,52,53].

Similar to patients with PKR2 mutations, PKR2−/− mice exhibit hypoplasia of the olfactory bulb, HH and the absence of GnRH neurons in the hypothalamus. PKR2 is indeed involved in the development and migration of GnRH neurons and its absence leads to an accumulation of neural progenitor cells, resulting in a reduced number of GnRH neurons in the hypothalamus and a lack of GnRH secretion associated with low plasma levels of the follicle-stimulating hormone (FSH) and impaired sexual maturation [54,55]. Similarly, PK2−/− mice do not reach puberty, and have low gonadotropin levels and a marked decrease in hypothalamic GnRH neurons. Interestingly, the HH phenotype in mice was only observed in homozygotes with PK2 deletion, suggesting that one normal allele can compensate for the inactivity of the other [54,55].

### 4.2. PK2 Mutations

Five mutations were identified in the PK2 gene. The PK2 mutations p.C34Y and p.R73C prevent the correct formation of disulfide bonds. The p.I79fsX1 and p.I79fsX1 are frameshift mutations that introduce a premature stop codon, resulting in the production of a non-functional truncated protein [56,57,58]. More recently, a new mutation has been identified at the intron–exon boundary (c.223-4C>A) that prevents recognition by the spliceosome complex and leads to the synthesis of a truncated protein encoded by the first two exons and part of the second intron (192 bp). Interestingly, by binding this mutated sequence, miR-3195 is able to promote the correct splicing of the PK2 transcript. In other words, the authors propose that miR-3195 is a natural therapeutic target that recognises this particular PK2 polymorphism [59].

### 4.3. PKR2 Mutations

Several different PKR2 mutations associated with KS/HH have been identified [56,60,61,62].

Six mutations (R80C, L173R, W178S, G234D, V274D and P290S) have been reported to affect trafficking. The mutants L173R, G234D, V274D and P290S were analysed to investigate the rescuing effect of a small molecule PKR2 antagonist, A457. The results show that A457 can rescue the expression and cell surface localisation of the P290S and V274D mutants, but not that of the W178S and G234D mutants. This result was explained by the hypothesis that A457 can only affect the conformation around its binding site near the fourth transmembrane domain, in the region adjacent to residues P290 and V274 [63]. It has been shown that the P290S mutant does not reach the membrane because it is recognised by a control mechanism of the post-endoplasmic reticulum (post-ER). This is a second control process that takes place in the Golgi and only considers proteins that have already passed the ER quality control system check. The post-ER determines whether proteins can be transported to the cell surface or instead return to the ER for degradation [64].

The characterisation of two of the PKR2 mutants identified in KS, S202G and Q210R, confirmed the central role of PKR2 ECL2 (Extracellular loop) in ligand binding [60,61]. This has already also been suggested by computational analyses [65,66] and by the demonstration of the crucial role of tryptophan 212 for PK2 binding [67]. Finally, the mutations R85C, R85H, R164Q, R268C, V331M, L218P and R270H alter the G-protein-coupling of the receptor. These are distorted mutations that alter one signalling pathway more than the other [62,68,69,70,71].

To characterise the mutants based on their ability to act in heterozygosity, the wild-type (WT) and mutant receptors were co-expressed in HEK-293 cells. Several of the mutant receptors tested did not interfere with cell surface targeting of the WT receptor. This argues against a dominant negative effect of the mutations in vivo [60,70]. A single R80C mutation of PKR2 leads to a significant reduction in receptor activity and exerts a dominant negative effect on WT PKR2 in vitro [61]. A further study investigated whether the Kallmann mutants are still able to recruit β-arrestins after ligand activation. The results show that four PKR2 receptor mutants (R85C, R85H, R164Q and V331M) were able to correctly recruit β-arrestins. In contrast, the R80C receptor was able to activate the three types of G proteins but was unable to recruit β-arrestins [69].

Several founding mutations associated with HH are already known. L173R PKR2 was the most common founder mutation, occurring in 2.4% of unrelated HH patients of various ethnicities, including Caucasian, Mexican, Brazilian and Maghrebi. L173R PKR2 is the oldest mutation associated with HH with an estimated age of about 9000 years. The constant presence of this founder mutation in the human genome, despite its deleterious effects in the homozygous state, may be due to the “overdominant” selection of the heterozygous state [72]. It is hypothesised that L173R heterozygotes have a selective advantage in terms of protection against *T. cruzi* infection [73].

In a recently published paper, a very high frequency (13.3%) of PKR2 mutations was detected in Chinese patients with HH. The mutants studied showed that the lack of PKR2-associated cell migration was mainly due to disruption of the MAPK/ERK signalling pathway [74]. We have reported only the most representative KS mutations of the PK2 and PKR2 genes, but a complete review of all PK2/PKR2 mutations identified in patients with KS has been published by Sugisawa et al. [55]. Idiopathic central precocious puberty (CPP) is caused by premature activation of hypothalamic GnRH secretion without congenital or acquired organic lesions in the central nervous system (Table 1).

In 2017, Fukami et al. performed a comprehensive molecular analysis using next-generation sequencing and identified a novel heterozygous frameshift mutation in the PKR2 gene in a 3.5-year-old girl with CPP. The mutant has a deletion of the C-terminal region and consists of only the first five transmembrane elements [75]. The in vitro test showed that this receptor variant exerts no activity per se, while it increases the activity of the WT receptor in the heterozygous state. This result confirms previous studies that used FRET to show that a receptor consisting of only the first five transmembrane elements (TM1–5) is able to dimerise with the WT receptor and that the heterodimer has greater functionality than the homodimer of the WT receptor [76,77]. Subsequent molecular screening of girls with onset of CPP before the age of six revealed a statistically significant difference in the Mutation Annotation Format (MAF) of the polymorphism rs3746682, which is a synonymous Single Nucleotide Polymorphism (SNP) and probably plays no pathological role [78]. However, this variant has also been associated with methamphetamine dependence in the Japanese population [44] and bipolar disorder in Japanese women [29].

### 4.4. Congenital Anosmia

Congenital anosmia is a rare condition defined by the absence of the sense of smell from birth. Complete loss of the sense of smell is quite common and affects 5% of the population. However, only 1 in 10,000 people have a complete loss of smell from birth. Congenital anosmia is a phenotypic phenomenon that occurs in numerous pathologies such as KS, CHARGE syndrome, Bardet–Biedl syndrome and other syndromes [79]. A recent study has shown a phenotype due to mutations in the PKR2 gene that is characterised by congenital complete loss of olfactory bulb formation, but not associated with loss of reproductive function.

### 4.5. Pituitary Dysplasia

Isolated or multiple pituitary insufficiencies are rare disorders in which a deficiency of one, several, or all of the hormones produced by the pituitary gland results in hypopituitarism.

The aetiology of most cases of hypopituitarism remains unclear, although a number of genetic causes for isolated or multiple pituitary deficiencies have been identified in recent decades [80,81].

Hypopituitarism is associated with pituitary stalk interruption syndrome (PSIS) [82], which is characterised by an interrupted pituitary stalk, an absent or ectopic posterior pituitary lobe and hypoplasia or aplasia of the anterior pituitary [81,82,83]. Although PSIS is heterogeneous both in its magnetic resonance imaging (MRI) appearance [84] and in its clinico-biological presentation, it can often result in isolated growth hormone (GH) deficiency or multiple hypothalamic–pituitary (HP) deficits. Patients with PSIS have an increased risk of morbidity and mortality and, unlike patients with isolated GH deficiency, often suffer from breech presentation, hypoglycaemia and micropenises.

In fact, only three abnormal genes (HESX1, LHX4, SOX3) have been shown to be involved in PSIS, in no more than 3% of cases overall [83].

Recently, PKR2 [85] has been reported in rare cases of PSIS. The ligand PK2 and its receptor PKR2 are involved in endocrine angiogenesis and neuronal migration and are expressed in the hypothalamus and pituitary gland [1,52,84]. Mutations in the PK2 and PKR2 genes in humans with HH have been associated with variable extrahypophyseal malformations [52]. These data led the researchers to speculate that the PK system might play a role in PSIS. For this reason, Raynaud and colleagues [85] screened 72 hypopituitarism patients with PSIS for the PK2, PKR2, LHX4, OTX2, SOX3 and HESX1 genes and identified three allelic variants of PKR2 associated with PSIS. Two of these variants, PKR2 mutations, are p.Arg85His and p.Leu173Arg. They have been previously identified in HH and have been shown to be detrimental to protein function, suggesting a causative role in this phenotype [58,60]. The third allelic PKR2 variant, p.Ala51Thr, previously described as a rare missense PKR2 variant [51], was found in non-consanguineous familial cases, with two affected sisters and their asymptomatic mother carrying the allelic variant. In vitro studies performed to evaluate the functional effects of these variants did not confirm deleterious effects on the Ca2 +-sensitive Gq protein pathway as well as other pathways linking prokineticin to Gi or Gs proteins, which partly explains the low correlation between genotype and phenotype. The prevalence of phenotypically relevant PKR2 mutations in the 72 patients analysed was 2.8%, suggesting a possible digenic or oligogenic inheritance in families with combined pituitary hormone deficiency (CPHD) with heterozygous PKR2 mutations, as previously described in HH patients [52].

In 2015, a novel heterozygous PKR2 variant (c.742C>T; p.R248W) was identified in a patient with CPHD, morning glory syndrome and a severely malformed pituitary gland with hormone deficiency and optic nerve malformation by targeted next-generation sequencing to search for the genetic causes of combined pituitary hormone deficiency (CPHD) [86]. The substituted amino acid was localised in the third intracellular loop of PKR2 and was found to be detrimental to receptor function. Indeed, one patient with the p.R248W PKR2 mutation, inherited from the patient’s mother, had slightly delayed menarche. These data provide further genetic evidence of a link between heterozygous PKR2 mutations and the development of CPHD.

A recently published paper [87] investigated the role of the PKR2 gene in the aetiology of multiple pituitary hormone deficiency (MPHD) and isolated growth hormone (GH) deficiency.

Using a candidate gene approach, 59 patients with MPHD, GH deficiency and idiopathic short stature were analysed. In four of these patients, two PKR2 deficiencies were found in heterozygous form: Leu173Arg and Arg85His. Two patients had short stature and were diagnosed with GH deficiency; the other two patients had central hypothyroidism and cryptorchidism and were diagnosed with MPHD. Expression variations or lack of penetrance in heterozygous carriers may indicate oligogenic inheritance or other environmental modifiers.

## 5. Disease of the Reproductive System

During ovulation, the follicle is expelled from the ovaries and transported through the fallopian tubes into the uterus, where it transforms into the corpus luteum in response to the LH surge. During this phase, known as the luteal phase, the secretory cells of the follicle are transformed into cells that primarily secrete progesterone, which promotes changes in the lining of the uterus that are essential for implantation of the embryo and maintenance of the pregnancy in the first trimester. Shortly after implantation, the blastocyst forms into the placenta, a temporary embryonic and later foetal organ that plays a fundamental role in facilitating the exchange of nutrients, gases and waste products between the maternal and foetal circulations. The placenta is also an important endocrine organ that prolongs the life of the corpus luteum through the secretion of human chorionic gonadotropin (hCG).

**Figure 4:**
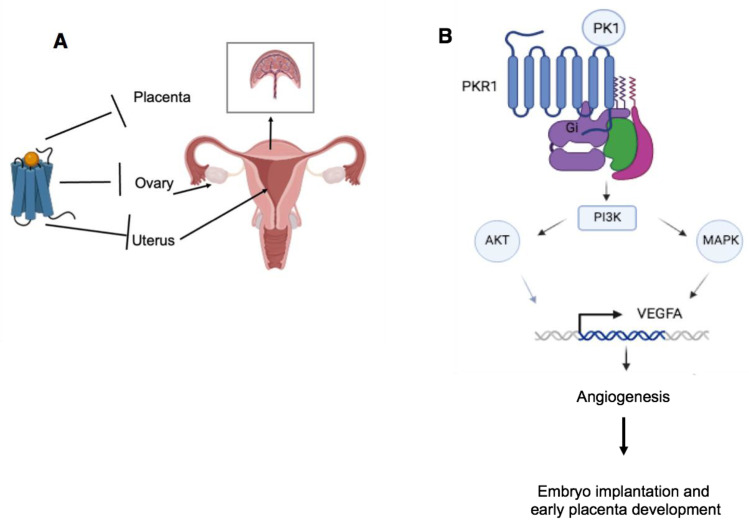
Female reproductive system. (**A**). The presence of polymorphisms in the genes of the prokineticin system determines the dysfunction of the placenta, ovaries and uterus, which leads to pregnancy failure. (**B**). Regulation of VEGFA gene expression by the prokineticin system.

Prokineticins and their receptors are expressed in various tissues of the female reproductive system [88,89] (Figure 4).

The PKR1 and PKR2 receptors are constantly present in the ovary, but the expression of PK1 is finely regulated during follicular maturation in the uterus and is inversely related to the expression of vascular endothelial growth factor (VEGF) [90]. A high expression of PK1 and a low expression of VEGF is found particularly in primordial, primary and secondary follicles. In the antral follicle, on the other hand, PK1 is only expressed in small amounts in the theca cells, while VEGF expression is very high in the granulosa cells and moderate in the theca cells. Finally, in the mature atretic follicle, PK1 expression is also strong in the remaining theca cells, while VEGF expression is weak [91].

In the uterus, PK2, PKR1 and PKR2 are expressed in the endometrium but show no temporal fluctuations in their expression during the menstrual cycle. In contrast, PK1 is only expressed at reproductive age and expression varies during the menstrual cycle, with low expression in the early follicular phase, followed by a gradual increase in expression, peaking in the mid-luteal phase, and finally a decrease in expression in the late luteal phase. This differential expression of PK1 is hormonally modulated by oestrogen and progesterone [92,93,94].

PK1 and PKR1 are also expressed in the placenta, where they play an important role in the processes of implantation, invasion and angiogenesis [95,96,97,98,99].

A peak in their expression is observed during the crucial hypoxic period of the placenta (8–10 weeks) before the haemochorial circulation is established. Hypoxic regulation of PK1 and PKR1 is mediated by hypoxia-inducible factor (HIF-1α), which recognises the promoters of PK1 and PKR1, but also by factors such as human chorionic gonadotropin and progesterone [100].

PK1 plays a pleiotropic role in trophoblast function during early placentation by stimulating PI3K/AKT/mTOR, MAPK-mediated cell proliferation and embryo adhesion mediated by MAPK and/or PI3K/AKT signalling pathways. The effects of PK1 on foetal endothelial cell growth are associated with PKR1, while activation of PKR2 promotes transcellular permeability [101]. In addition, PK1 also appears to be involved in the success of embryo implantation by modulating the expression of key genes involved in pro-implantation processes, including IL-11, IL-6, IL-8, LIF and COX-2 [102,103,104]. Finally, PK1 acts on the angiogenesis of the placental endothelial system [97,98,99].

Deregulations of PK1 signalling have been found in human ectopic endometriosis, human polycystic ovaries and pregnancy-related diseases such as pre-eclampsia, recurrent miscarriage, intrauterine growth restriction and preterm birth [105,106,107,108,109,110].

Recurrent pregnancy loss (RPL) is a highly multifactorial condition due to the deregulation of key genes involved in pregnancy. However, due to the multifactorial aetiology, complex features and different prevalences of genetic variants in different populations, it is extremely difficult to identify risk factors or molecular markers [111].

Recently, genetic variants of PK1, PKR1 and PKR2 have been shown to confer susceptibility to RPL in a Taiwanese Han population. Multifactor dimension reduction (MDR), a nonparametric data reduction method, was applied to identify multilocus genotype combinations for predicting disease risk. Although PK1 polymorphisms per se did not show significant differences between patients and controls, MDR analysis revealed significant genetic interactions between three loci (PK1, PKR1 and PKR2), and these three loci jointly confer susceptibility to RPL [112]. The analysis of the association of PKR1 rs4627609 and PKR2 rs6053283 polymorphisms with idiopathic RPL was also investigated in the Han population of eastern China. The results obtained confirmed the association between PKR2 rs6053283 and RPL, but not the association between PKR1 rs4627609 and RPL. These inconsistent results may be mainly attributed to the small samples of both reports and the differences in geographical environments between Taiwan and eastern China [113].

The PKR1 I379V (SNP rs347157) and PKR2 V331M (SNP rs5282731) variants, which confer a lower risk of RPL and may play a prophylactic role in early pregnancy by altering calcium signalling and facilitating cell invasiveness, were also identified in a Taiwanese Han population [114].

The mutation V67I (SNP rs7514102) of PK, which does not cause changes in the functionality of the protein but decreases the efficiency of gene expression, was also identified. The mutant is therefore able to promote trophoblast invasion in a dose-dependent manner similar to the WT protein. However, in the presence of the mutated gene, a reduction in cellular invasiveness and PK1 protein production is observed [115] (Table 1).

PK1 and the vascular endothelial growth factor A (VEGFA) system cooperate in pregnancy-associated angiogenesis by regulating key signal transduction pathways such as Akt, IL-8, EGFR, MAPK, SRC, VHL, HIF-1A and STAT3. Dysregulation of these processes has been associated with RPL. Polymorphisms of the PKR2 gene (V331M) and the VEGFA receptor gene [KDR (Q472H)] have been shown to be significantly associated with idiopathic RPL [116,117].

## 6. Disease of the Digestive System

The enteric nervous system (ENS) is located in the gastrointestinal tract and consists of a large number of nerve cells organised in interconnected ganglia that arise from neural crest cells (NCCs) during embryogenesis. Failure of these processes leads to aganglionic conditions of the colon, also known as Hirschsprung’s disease (HSCR), which is the main cause of functional intestinal obstruction in newborns. PK1 and PK2 as well as PKR1 and PKR2 are present in human intestinal NCKs. Through mutational analysis of the genes encoding the prokineticin system, we identified the following mutations in a cohort of HSCR patients: one R48W mutation (SNP rs62623571) in PK1, one K354N mutation in PKR1 and six mutations in PKR2: G68S, R85C, R85H, R268C (SNP rs78861628), P290S, Y297I. This analysis suggests that these genes can be considered disease susceptibility genes [118,119,120] (Table 1).

## 7. Obesity

A combination of environmental and genetic factors influences the development of obesity. Advances in genomics have led to the identification of several genetic loci associated with this disease [121]. The PK2 mutations identified in KS are associated with an obese phenotype. Moreover, the dysregulation of the prokineticin system in obesity could be due to the presence of mutations in the accessory PKR protein, the accessory melanocortin 2 receptor protein (MRAP2) [122]. Indeed, variants of MRAP2 have been identified that increase the risk of obesity [123,124].

**Figure 5:**
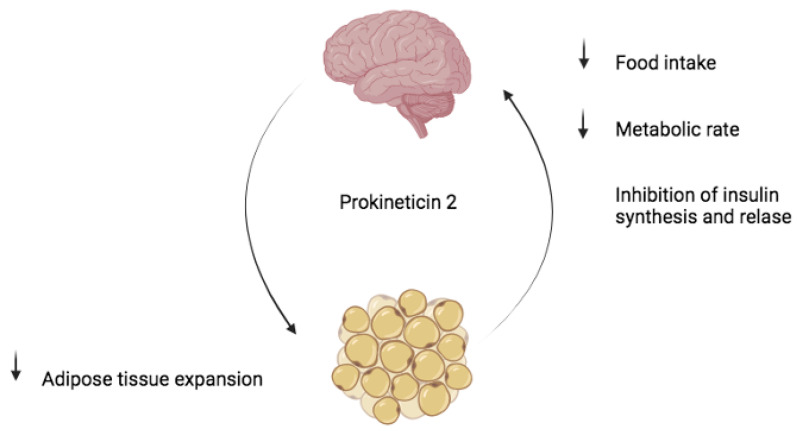
Prokineticin-2 controls food intake and fat tissue expansion [127,128].

Obese people often have poor sleep quality, and at the same time, poor sleep quality promotes overeating. PK2 has been shown to be involved in the signalling mechanism underlying the reciprocal control of appetite (Figure 5) and sleep [125,126,127]. Specifically, neurons expressing PKR2 in the parabrachial nucleus (PBN) receive direct projections from neurons expressing neuropeptide Y receptor Y2 (NPY2R) in the lateral preoptic area (LPO) of the hypothalamus. Genetic silencing of PKR2 signalling in PKR2-PBN neurons reduces hyperphagia, weight gain, and sleep disturbances in obese mice [128].

## 8. Conclusions

The presence of the prokineticin system in different cell types, its conservation in the evolutionary scale [129,130] and its ability to fulfil various essential functions within the organism underlines the importance of the prokineticin system and prompted us to study, in depth, the molecular mechanisms underlying its functions with the aim of identifying new targets that can be used as drugs.

## Figures and Tables

**Table 1:** Polymorphisms in prokineticin receptors and ligands in miscellaneous diseases.

Gene	Polymorphism	Disease	References
**PK1**	rs 62623571	HSCR	Ruiz-Ferrer et al., 2011
	rs7514102	RPL	Su et al., 2016
**PK2**	rs1316780 rs10865660 rs3796224 rs1374913	MDD, BP	Kishi et al., 2009
	rs75433452	MUD	Jiang et al., 2023
**PKR1**	rs4627609 rs347157	RPL	Su et al., 2010 Cao et al., 2016
**PKR2**	rs17721321 rs6085086 rs3746684 rs3746682 rs4815787	MDD, BP	Kishi et al., 2009
	rs6085086 rs3746682 rs4815787	MUD	Guerina et al., 2021.
	rs3746682	CPP	Aiello et al., 2021
	rs6053283 rs5282731	RPL	Su et al., 2010 Cao et al., 2016
			Su et al., 2013; Su et al., 2014
	rs78861628	HSCR	Rui-Ferrer et al., 2011

Abbreviations: HSCR = Hirschsprung’s disease; RPL = recurrent pregnancy loss; MDD = major depressive disorder; BP = bipolar disorder; MUD = methamphetamine use disorder; CPP = idiopathic central precocious puberty.

## Data Availability

No new data were created or analyzed in this study. Data sharing is not applicable to this article.
